# Allometric Modeling of Wingate Test among Adult Male Athletes from Combat Sports

**DOI:** 10.3390/medicina56090480

**Published:** 2020-09-21

**Authors:** Manuel J. Coelho-e-Silva, Paulo Sousa-e-Silva, Vinícius S. Morato, Daniela C. Costa, Diogo V. Martinho, Luís M. Rama, João Valente-dos-Santos, André O. Werneck, Óscar M. Tavares, Jorge Conde, Joaquim M. Castanheira, Rui Soles-Gonçalves, João P. Duarte

**Affiliations:** 1 Faculty of Sports Science and Physical Education, University of Coimbra, CIDAF (uid/dtp/04213/2020), 3040-248 Coimbra, Portugal; paulomrss@hotmail.com (P.S.-e.-S.); danicrava@hotmail.com (D.C.C.); dvmartinho92@hotmail.com (D.V.M.); luisrama@fcdef.uc.pt (L.M.R.); j.valente-dos-santos@hotmail.com (J.V.-d.-S.); joaopedromarquesduarte@gmail.com (J.P.D.); 2 Department of Physical Education, University of Coimbra, FCDEF, 3040-248 Coimbra, Portugal; taz_morato@hotmail.com; 3 Faculty of Physical Education and Sport, Lusofona University of Humanities and Technologies, 1749-024 Lisbon, Portugal; 4 São Paulo State University “Júlio de Mesquita Filho” (UNESP), Presidente Prudente 19060-900, Brazil; andreowerneck@gmail.com; 5 Polytechnic Institute of Coimbra, Coimbra Health School, 3046-854 Coimbra, Portugal; oscar@estescoimbra.pt (Ó.M.T.); jconde@estescoimbra.pt (J.C.); jmc@estescoimbra.pt (J.M.C.); ruigoncalves@estescoimbra.pt (R.S.-G.)

**Keywords:** scaling, anaerobic fitness, air displacement plethysmography, dual energy X-ray absorptiometry (DXA), short-term maximal intensity

## Abstract

*Background and objectives:* Athletes from combat sports are grouped into a series of weight categories that are intended to promote fair competition. Differences in performance are partly attributable to differences in body size. Consequently, ratio standards in which a performance variable is simply divided by an anthropometric characteristic such as body mass are often used, although this application is not recommended. This study aimed to obtain allometric models to interpret Wingate Anaerobic Test (WAnT) outputs among male adult athletes from combat sports. *Materials and Methods:* The sample was composed of 64 participants aged 18–39 years (24.2 ± 4.6 years). Stature and body mass (BM) were measured and air displacement plethysmography used to estimate fat mass and fat-free mass (FFM). Lower-limb lean soft tissue (LL-LST) was derived from dual energy X-ray absorptiometry. WAnT outputs were peak power (WAnT-PP) and mean power (WAnT-MP). Allometric models were obtained from simple and multiple linear regressions using log-transformed variables. *Results:* Models derived from a single three-dimension descriptor explained a large portion of variance: WAnT-PP (BM: 31.1%; FFM: 54%; LL-LST: 47.2%) and WAnT-MP (BM: 50.1%; FFM: 57.4%; LL-LST: 62.7%). Finally, the best proportional allometric models emerged from the combination of LL-LST and FFM (WAnT-PP: 55%; WAnT-MP: 65%). *Conclusions:* The relationship between weight categories and performance did not seem to be explained by the basic principles of geometric similarity.

## 1. Introduction

Combat sports include combinations of movement by the whole body and limbs (upper and lower). The pattern of effort is characterized by multiple high-intensity episodes of short duration (rounds) interspersed by intervals that, depending on the specific combat sport and specific regulations, can range from seconds to minutes. Meantime, anaerobic metabolism refers to the ability to produce energy by intramuscular phosphates (ATP and PCr) and/or anaerobic glycolysis during a specific type of short-duration exercise [1]. Direct measurement of substrate utilization and of the amount of energy obtained by anaerobic metabolism during whole-body exercise is not practicable. In the absence of intra-muscular data, research on anaerobic performance has focused on the assessment of either the rate or the amount of the mechanical energy yield [2], in the context of maximal-intensity protocols that are performed ‘all-out’.

There is a variety of procedures for assessing the ability to perform maximal-intensity exercise. Cycle ergometer tests, particularly the Wingate Anaerobic Test (WAnT), are often used in the literature, including that on combat sport [3,4,5]. The WAnT protocol was developed at the Wingate Institute [6]. Since then, it has been refined and a comprehensive description is available elsewhere [7,8]. Participants have to pedal flat out for a 30-s period on a cycle ergometer against an external load equivalent to 7.5% of body mass for Monark-type ergometers and 4% on Fleisch systems [6,7,8]. WAnT scores were originally subdivided into 5-s blocks with peak power output (in watts) supposed to occur in the first 5 s. The average over the 30-s is termed mean power. The construction of ratio standards, in which a performance variable is simply divided by an anthropometric size descriptor (such as body mass), is often used, although it may distort the interpretation of the data under investigation.

Profiling the physiological characteristics of athletes from combat sports who are officially matched by weight categories should not adopt the traditional approach of expressing watt per unit of body mass. Available evidence has demonstrated that body size was still correlated to physiological performance even when it was expressed per unit of body mass [9,10]. The use of ratios tends to penalize individuals having a large body size and, consequently, simple ratios are not an effective tool to remove the influence of body mass from maximal performance outputs [11].

“All-out” exercise is determined by the demands of metabolic active tissues and, thus, other size descriptors should be systematically considered as an alternative to body mass for the correct interpretation of inter-individual variance in WAnT performance. Indeed, recent studies suggested fat-free mass (FFM) and appendicular volume as relevant descriptors to normalize short and intermediate maximal outputs [12]. Stature is a linear variable and consequently considered a one-dimensional body size descriptor. Meantime, masses (whole body and fat-free) and volume move towards the assumption of a three-dimension descriptor.

Athletes in combat sports often tend to manage their body mass in order to experience competitions in various weight categories. In the context previously stated, inter- and intra-individual weight-related variation in performance needs to be adequately interpreted. Allometric scaling appears as a valid statistical approach to create a ‘size-free’ expression of performance [11,12,13,14,15]. In this sense, allometry is viewed as an adequate approach in the interpretation of Wingate outputs without ignoring variation in body size. Proportional allometry combining more than one body size descriptor minimizes the impact of heteroscedastic (non-constant variation) errors and provides more plausible interpretations compared to models based on single size descriptors [14,15].

The current study aimed to identify the best body size descriptors to obtain size-independent scaling exponents for the interpretation of the Wingate Test outputs (peak power: WAnT-PP; mean power: WAnT-MP) among male adult athletes in combat sports. It was hypothesized that body size would be directly correlated to performance. In addition, it was also hypothesized that an appendicular descriptor (independently and combined) can account for inter-individual variance in WAnT performance.

## 2. Materials and Methods

### 2.1. Procedures, Ethics Statement and Participants

The research project was approved by the Ethics Committee (Faculty of Sports Science and Physical Education of the University of Coimbra: CE/FCDEF-UC/00102014 informed at 31/03/2015). All procedures were in accordance with the ethical standards for sports medicine. Athletes were recruited after formal contacts with respective federations, clubs and coaches from different combat sports. Participants were informed about the objectives, procedures, the voluntary nature of their participation and that they could withdraw from the study at any time. They were instructed not to eat for at least three hours and not to drink coffee or beverages containing caffeine for at least eight hours before testing. Prior to data collection, written informed consent was obtained from each individual participant in accordance with the Declaration of Helsinki. Participants visited the laboratory, and data collection started at the same hour of the day (8:00 a.m.) following the same sequence: anthropometry, air displacement plethysmography (ADP), dual energy X-ray absorptiometry (DXA) and WAnT. All participants were familiar with the battery of tests including protocols using the cycle-ergometer. Assessments were completed by experienced technicians adopting standardized procedures. The sample was composed of 64 male athletes aged 18–39 years (24.2b ± 4.6 years): their combat sports were kickboxing (n = 10), Jiu-jitsu (n = 10), judo (n = 10), boxing (n = 11), taekwondo (n = 9), wrestling (n = 5), karate (n = 9). Inclusion criteria were as follows: registered in respective national federations, at least two years of previous experience in competitive combat sports (9 ± 6 years of training), participation in national or international competitions. Exclusion criteria were: time loss due to injury during the previous eight weeks; involved in weight-loss interventions in the previous eight weeks.

### 2.2. Anthropometry

Anthropometry was measured by a single observer following standardized procedures [16]. A portable scale was used to measure body mass (Seca model 770, Hanover, MD, USA) to the nearest 0.1 kg. Stature was measured to the nearest 0.1 cm using a stadiometer (Harpenden model 98.603, Holtain LTD, Crosswell, UK). A subsample of 13 adult male athletes was measured twice to allow the determination of the technical error of measurement (TEM). The data quality has already been reported elsewhere [17]. Expressed in the same units as measured, the following information was calculated: stature (TEM = 0.37 cm) and body mass (TEM = 0.56 kg).

### 2.3. Air Displacement Plethysmography (ADP)

ADP technology (Bod Pod Composition System, model Bod Pod 2006, Life Measurement, Inc, Concord, CA, USA) was used to assess body volume to the nearest 0.001 L with participants using Lycra underwear, a swim cap and without shoes as recommended by the manufacturer. Before each trial, the equipment was calibrated using a cylinder of 50.255 L with the observer adopting the instructions of the manufacturer. Consecutive trials were performed for each participant who quietly sat in the chamber while raw body volume was repeatedly measured until two values within 150 mL were obtained. If more than three body volumes were needed, measurements were re-started after completing another calibration procedure. Thoracic gas volume was individually estimated and used to obtain body volume. Body density (kg∙L^−1^) corresponds to body mass (kg) divided by body volume (L) and permitted the determination of fat mass and fat-free mass using an equation recommended for normal-weight adults [18].

### 2.4. Dual-Energy X-ray Absorptiometry (DXA)

Absorptiometry (fan-beam Lunar DPX-PRO) was used to obtain estimates of fat and lean soft tissues. The calibration was checked and passed on a daily basis using the Lunar calibration phantom. Participants were placed in the supine position on the scanning table with the body aligned along with the central horizontal axis. Lower limbs were fully extended and feet were secured with a canvas and Velcro support to avoid foot movement during the scan acquisition. A single certified technician performed all scans and extracted the data following the guidelines published by the manufacturer (Lunar Encore software, version 13.6). The DXA reports included lean soft tissue, and fat tissue for the whole body and its parts (subhead, trunk, upper limbs, lower limbs). Lower limbs lean soft tissue (LL-LST) was retained for analysis

### 2.5. Wingate Anaerobic Test (WAnT)

While in the laboratory athletes were assessed for anthropometry, ADP and DXA. Afterwards, they completed a standardized 4-min warm-up on the cycle-ergometer (Monark 894 Peak Bike, Monark AB, Varberg, Sweden). The warm-up protocol included three maximal sprints for 3–4 s at the end of each minute. Subsequently, static stretches of the quadriceps and hamstring and abductors muscles were performed under supervision. The 30-s WAnT protocol requires a standardized resistance of 7.5% of body mass [7,8] and is described elsewhere [12]. The participant cycled at 60 rpm (revolutions per minute) and started after a verbal signal (3-2-1-Go). The braking force was automatically applied when 70 rpm was exceeded. During the test, participants were seated and used toeclips. They received standardized verbal encouragement. The ergometer used a sampling rate of 50 Hz. Performance outputs were extracted (in watts) using Anaerobic Test Software (Monark Exercise AB, Vansbro, Sweden). The following parameters were retained for analysis: WAnT-PP (corresponding to the highest value observed during the initial 10 s) and WAnT-MP (defined as the average performance during the test).

### 2.6. Data Analysis

Descriptive statistics were determined for the total sample and the Kolmogorov-Smirnov test examined the normality for each variable. Pearson correlation coefficients were used to examine the linearity between body size descriptors (stature, body mass, FFM, and LL-LST) and WAnT outputs (WAnT-PP, WAnT-MP). Simple allometric models were obtained using Equation (1). For each of the WAnT_i_ outputs, for X_i_ size descriptors (mentioned above) with a magnitude of correlation coefficient classified as moderate, large or very large, allometry was explored. Values of “a” and “k” were derived from linear regressions of the logarithmic transformations as summarized in Equation (2). Log (WAnTi) corresponds to the natural logarithms of the physiological variable under analysis, “a” was the constant, and “k” the scaling coefficient for each size descriptor (natural log-transformed). Validation of each allometric model was examined by the inspection of the association between scaled performance variables and the respective size descriptor. Stepwise multiple linear regression on log (WAnT_i_) were performed based on proportional allometric models [14,15] aiming to incorporate more than one body size descriptor as presented in Equation (3). For this purpose, only the best model for each WAnT output was retained for discussion.

Equation (1):

(1) $${\mathrm{WAnT}}_{i}=a\;.{\;X}_{i}^{k}.\;\varepsilon$$

Equation (2):

(2) $$\log\;({\mathrm{WAnT}}_{i})=\log\;\left(a\right)+k_{1}.\;\log\;(X_{1})+\;\log\;\left(\varepsilon \right)$$

Equation (3):

(3) $$\log\;({\mathrm{WAnT}}_{i})=\log\;\left(a\right)+k_{1}.\log\;(X_{1})+{\;k}_{2}.\log\;(X_{2})+\;\log\;\left(\varepsilon \right)\;$$

The magnitude of correlations was interpreted as follows [19]: trivial (r < 0.1), small (0.1 ≤ r < 0.3), moderate (0.3 ≤ r < 0.5), large (0.5 ≤ r < 0.7), very large (0.7 ≤ r < 0.9) and nearly perfect (r ≥ 0.9). R^2^ provides an indication of the explained variance achieved by the independent variable(s) for each allometric model. Statistical significance was set at 5%. Analyses were performed using IBM version 24.0 software (SPSS Inc, Company, New York, NY, USA).

## 3. Results

**Figure 1:**
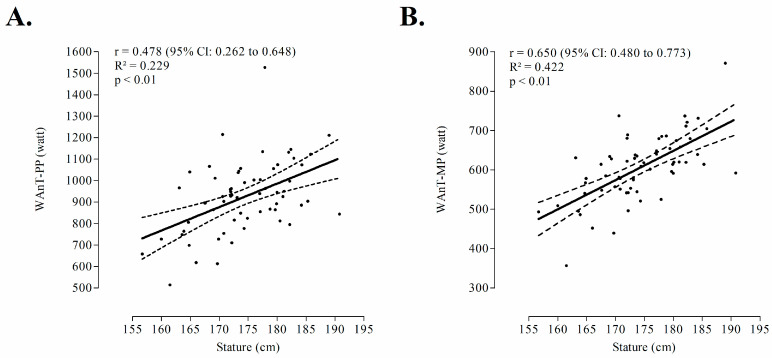
Correlation between one-dimensional size descriptor (stature), peak power (WAnT-PP, panel **A**) and mean power (WAnT-MP, panel **B**) outputs extracted from Wingate anaerobic test among male adult athletes in combat sports (n = 64)

**Figure 2:**
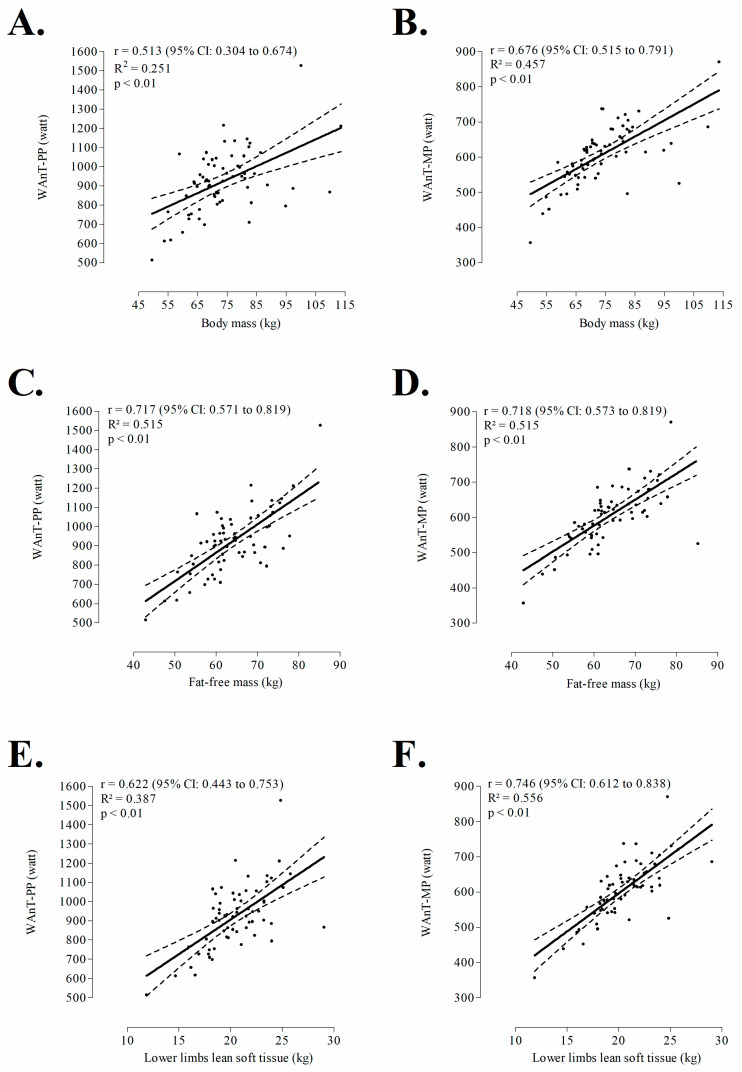
Correlation between three-dimensional size descriptors (body mass: panel **A** and **B**, fat-free mass: panel **C** and **D**, lower limbs lean soft tissue: panel **E** and **F**) and outputs extracted from Wingate anaerobic test among adult athletes of combat sports (n = 64).

Characteristics of the sample are summarized in Table 1. WAnT-PP was moderately correlated to stature (r = +0.478), largely correlated to body mass (r = +0.513) and LL-LST (r = +0.622) and, finally, very largely correlated to whole-body FFM (r = +0.717). In parallel, WAnT-MP was largely correlated to stature and body mass (coefficients ranged from +0.650 to +0.676) and very largely correlated to descriptors of metabolic active tissue: FFM (r = +0.718), LL-LST (r = +0.746), as presented in Figure 1 and Figure 2.

Simple allometric models for the WAnT outputs are presented in Table 2. Explained inter-individual variance ranged from 26.1% (stature) to 54% (FFM) for WAnT-P. In parallel, for WAnT-MP, explained variance was large for all size descriptors and ranged from 42% (stature) to 62.7% (LL-LST). As presented in Table 2, WAnT outputs established a linear relationship with whole body FFM (WAnT-P: k = 1.045; 95% CI: 0.801 to 1.290; WAnT-MP: k = 0.840; 95% CI: 0.656 to 1.8023). Stature consistently established a quadratic power function with the two performance parameters (WAnT-PP: k = 2.236; WAnT-MP: k = 2.206) while body mass consistently presented a scaling exponent lower than the unit (WAnT-PP: k = 0.643; WAnT-MP: k = 0.635). Finally, LL-LST suggested a linear relationship with WAnT-PP (k = 0.868; 95% CI: 0.635 to 1.101) and a non-linear relationship with WAnT-MP (k = 0.779; 95% CI: 0.627 to 0.932). All extracted allometric models appeared uncorrelated with respective size descriptors (correlation coefficients fluctuated between −0.011 and −0.041) suggesting that scaled performance outputs were effective in producing size independent scores for WAnT scores.

Proportional allometric models combining more than one size descriptor are summarized in Table 3. Generally, combining two body size descriptors explained 55–65% of the WAnT outputs. For both dependent performance variables, the predictors were whole-body FFM combined with LL-LST. For WAnT-PP, the overall model corresponded to a very large and significant correlation (r = +0.742; R^2^ = 0.550). For mean power, the multiple correlation coefficient was +0.804 (R^2^ = 0.647).

## 4. Discussion

Given the relevance of anaerobic fitness to combat sports, this study examined the association between WAnT peak and mean power outputs and whole-body size descriptors plus appendicular lean soft tissue. The one-dimensional descriptor (stature) and the three-dimensional descriptors (body mass, FFM and LL-LST) were substantially correlated to performance variables, with larger coefficients obtained for the WAnT-MP compared to WAnT-PP. It was possible to obtain size-free models based on the list of descriptors that were at least moderately correlated with performance. The scaled variables emerged as independent from the respective descriptor. The results of the allometric models in this study identified FFM of the whole-body obtained from ADP as the most prominent contributor in explaining inter-individual variance for WAnT-PP, while for WAnT-MP the best single predictor was the LL-LST obtained from DXA. Derived from logarithmic transformations, almost all size descriptors were different from 1.0 corroborating a non-linear relationship between independent variables and maximal performance outputs [13,14]. The exception was an apparent quasi-linear relationship between WAnT-PP and FFM. However, this should be interpreted with caution given the large range of confidence intervals. Moreover, body mass established a consistent non-linear relationship with performance parameters. Finally, compared to simple allometric models, proportional allometry explained a larger amount of inter-individual variance for both WAnT outputs. Only the most prominent proportional models were interpreted, and they highlighted the relevance of considering both whole body (FFM) and appendicular (LL-LST) size descriptors that correspond to indicators of metabolic active tissues (fat-free mass components).

The means for stature and body mass of the total sample (174.3 ± 7.2 cm; 73.6 ± 12.1 kg) did not substantially differ from corresponding values reported for the general Portuguese population [20]: 173.2 ± 7.4 cm, 78.6 ± 11.6 kg. Table 4 summarizes descriptive statistics by sports in the current sample. Given the small sample size, comparisons between groups should be made with caution. Table 4 also includes descriptive statistics for chronological age, body size descriptors and body composition in addition to absolute and relative values (traditional ratios adopted by the original authors) for WAnT outputs in several studies of male adult athletes in different combat sports: karate [21], judo [22], boxing [4], taekwondo [23], kickboxing [24], wrestling [4,25] and JiuJitsu [26]. Mean differences in performance characteristics between studies probably reflect variation in body size between samples. Consequently, techniques to partition out body size have to be used. In this sense, traditional ratios have been used to mitigate the confounding effect of body size on WAnT performance [4,20,21,22,23,24,25].

It is now consensual that the ratio in which a performance variable is simply divided by a size descriptor (such as body mass) misleads the interpretation by distorting the data under investigation. Scaling, as it is called, is currently an area of renewed interest [27,28] although early considerations date back several decades [10]. Interpretation of WAnT power outputs in sports characterized by a broad inter-individual variability in body size, as is the case in combat sports whose competitors are matched according to weight categories, should be based on power function ratios [2,10,11,12,13,14,15] that are obtained from allometry.

The ability to perform short maximal efforts is considered a relevant characteristic in combat sports given the intermittent nature of the efforts required [26,28,29,30,31,32]. The 30-s WAnT has been reported as the most common method to evaluate peak and mean outputs [4,8,12,20,21,22,23,24,25]. A large variability among the sample in the current study was noted for performance outputs: 514 to 1527 watt (WAnT-PP) and 357 to 871 watts (WAnT-MP). In addition, within the sample of the current study, body mass presented a broad inter-individual range (49.5 to 113.5 kg). Fluctuations in body mass may have an impact on performance, but the magnitude of the associations seemed to vary according to the body component (tissue) that is fluctuating. The present study evidenced that a decrease of 1-kg in body mass was associated with a reduction of 6.99 watt in the WAnT-PP and 4.62 watt in the WAnT-MP, while a reduction of 1-kg in FFM was associated with a decline of 14.73 watts and 7.39 watts, respectively. Finally, a decrease of 1-kg of LL-LST predicted a decline of 35.99 watts of peak and 21.62 watt of mean WAnT outputs. In this sense, appendicular metabolic tissues should be interpreted as relevant contributors to anaerobic performance.

By inference, athletes in combat sports must monitor their body mass which defines the categories for competitions, preferably combined with the assessment of body composition, particularly fat-free mass, and the amount of appendicular mass. In the context of combat sports, many athletes have already experienced programs of sudden weight loss in order to compete in a weight category that intuitively gives them an advantage. However, adult humans are not supposed to be geometrically similar to each other [33,34]. The current study reported exponents that were near linearity (scaling exponents were 0.840 and 1.045 for FFM). In parallel, a power function of 2/3 (that is 0.643 or 0.635, respectively for WAnT-PP and WAnTMP) was found for body mass. Actually, FFM and LL-LST were the best single predictors of WAnT outputs reinforcing the hypothesis that active tissues were more adequate in order to normalize for inter-individual variation in body size [12]. This has obvious implications for weight loss programs that consider above all a decrease in FFM (mainly water). A group of boxers was submitted to a five-day body mass reduction, body fat percentage determined via bioelectrical impedance decreased by 0.5%, FFM by 4.9%, with most of the body mass reduction obtained via dehydration as decreases in total body water (6%), extracellular water (12.4%), total hemoglobin mass (5.3%), blood volume (7.6%) and plasma volume (8.6%) observed in the group submitted to the weight loss procedures [35].

The interpretation of current results should not ignore that sample size is too limited to permit a generalization to all athletes in combat sports. A greater sample stratified by weight categories considering each of the various combat sports is recommended for future studies. In addition, although a strong correlation between upper and lower limbs peak power was found among twelve male boxers, laboratory assessment of physical fitness and physiological profile of athletes in combat sports should include upper limbs WAnT [36]. Other protocols available in the literature reporting physical and physiological characteristics of athletes from combat sports include treadmill running to determine maximal oxygen uptake, maximal punching performance, hand grip strength, vertical jump, isokinetic strength, side step tests, and agility given by side step tests [5,37]. Despite the apparent correlation among WAnT protocols to assess upper and lower limbs power, it is relevant to remember that WAnT involves pedaling a cycle ergometer against a standardized braking force and, in this particular, the literature suggested a force-velocity test (FVT) as an alternative [38]. This is composed of three–five sprints performed against a range of braking forces (Fb) [2,9,27,38]. A parabolic relationship among Fb and power, in addition to a quasi-linear relationship between Fb and velocity, enable the individual determination of optimal velocity and optimal Fb to estimate optimized peak power for each participant. Actually, recreational athletes produced their maximal peak power values against an optimal load equal to 10% BM, while for high-level trained athletes the FVT appeared to be more appropriate in assessing peak power and few cases required Fb higher than 11% BM [39]. Note, however, that FVT does not provide an alternative to WAnT-MP which in combat sports is probably as relevant as WAnT-PP. Finally, due to the cross-sectional design of the current study, caution is needed to generalize the observed interrelationship among BM, FFM, LL-LST, WAnT-PP and Wan-MP for the purpose of intra-individual changes in body mass and its components.

## 5. Conclusions

In summary, allometric models were able to produce size-free exponents for WAnT performance outputs. Given the association between specific body components and anaerobic performance, body weight reductions should be critically managed, especially having in mind the avoidance of excessive losses in FFM. Future studies may investigate longitudinal association between body size descriptors and anaerobic performance. They should be aimed at investigating the intra-individual variability of decrements in performance associated with deliberate pre-competitive weight changes.

## Figures and Tables

**Table 1:** Descriptive statistics for anthropometry, body composition and Wingate Test outputs, normality test and correlation between size descriptors and performance outputs obtained from the Wingate Test among male adult athletes in combat sports (n = 64).

Variables (X_i_)	Unit	Range	Mean			Standard Deviation	Normality		Correlation (X_i_·Y*_i_*)			
		(min–max)	Value	SEM	(95% CI)		K-S Value	p	Y_1_: WAnT-PP		Y_2_: WAnT-MP	
									r	p	r	p
Anthropometry												
Stature	cm	156.7–190.8	174.3	0.9	(172.5 to 176.1)	7.2	0.064	0.20	0.478	≤0.01	0.650	≤0.01
Body Mass	kg	49.5–113.5	73.6	1.5	(70.6 to 76.7)	12.1	0.110	≤0.05	0.513	≤0.01	0.676	≤0.01
Air Displacement Plethysmography												
Body Volume	L	46.6–110.2	69	1.5	(66.0 to 72.1)	12.1	0.111	≤0.05				
Body Density	kg/L	1.013–1.093	1.071	0.002	(1.067 to 1.075)	0.016	0.144	≤0.01				
Thoracic Gas Volume	L	2.870–4.574	3.751	0.044	(3.664 to 3.839)	0.351	0.058	0.20				
Fat Mass	%	2.7–38.8	12.7	0.9	(10.9 to 14.4)	7.0	0.140	≤0.01				
	kg	2.2–42.4	9.9	0.9	(8.0 to 11.7)	7.4	0.212	≤0.01				
Fat-Free Mass	kg	42.9–85.1	64.0	1.01	(62.0 to 66)	8.1	0.090	0.20	0.717	≤0.01	0.718	≤0.01
Dual energy X-Ray Absorptiometry												
Lower Limbs Lean Soft Tissue	kg	11.9–29	20.5	0.4	(19.7 to 21.2)	2.9	0.072	0.20	0.622	≤0.01	0.746	≤0.01
Wingate Test												
Load (Braking Force)	kg	3.8–8.6	5.6	0.1	(5.3 to 5.8)	0.9	0.130	≤0.01				
WAnT-PP	watt	514–1527	923	21	(882 to 965)	166	0.070	0.20				
WAnT-MP	watt	357–871	606	10	(585 to 627)	83	0.069	0.20				

SEM (standard error of the mean); 95% CI (95% confidence interval); WAnT-PP (Wingate peak power output); WAnT-MP (Wingate mean power output).

**Table 2:** Single allometric modeling for the two functional outputs extracted from the Wingate anaerobic test using different body size descriptors among adult male athletes in combat sports (n = 64).

Y_i_: Performance Outputs	X_i_: Size Descriptor	log a + k × log (Size Descriptor) + log ε					Correlation (Y_i_/X_i_^k^, X_i_)	
		Intercept	k Exponent (95% CI)	r	R^2^	p	r	(95% CI) *
WAnT-PP	Stature	−4.725	2.236 (1.281 to 3.191)	0.511	0.261	<0.01	−0.018	(−0.262 to 0.228)
	Body Mass	4.057	0.643 (0.400 to 0.886)	0.558	0.311	<0.01	−0.031	(−0.274 to 0.216)
	FFM	2.473	1.045 (0.801 to 1.290)	0.735	0.540	<0.01	−0.011	(−0.256 to 0.235)
	LL-LST	4.200	0.868 (0.635 to 1.101)	0.687	0.472	<0.01	−0.015	(−0.259 to 0.231)
WAnT-MP	Stature	−4.990	2.207 (1.547 to 2.867)	0.647	0.420	<0.01	−0.016	(−0.260 to 0.230)
	Body Mass	3.675	0.635 (0.474 to 0.796)	0.708	0.501	<0.01	−0.041	(−0.283 to 0.206)
	FFM	2.912	0.840 (0.656 to 1.023)	0.758	0.574	<0.01	−0.019	(−0.263 to 0.227)
	LL-LST	4.053	0.779 (0.627 to 0.932)	0.792	0.627	<0.01	−0.024	(−0.268 to 0.223)

95% CI (95% confidence interval); WAnT-PP (Wingate peak power output); WAnT-MP (Wingate mean power output); FFM (fat-free mass); LL-LST (Lower limbs lean soft tissue); a (constant); k (beta exponent); r (Pearson correlation coefficient); R^2^ (coefficient of determination); * If 95% confidence interval crosses zero, the correlation coefficient is not significant.

**Table 3:** Proportional allometric modeling for the two outputs extracted from the Wingate Test combining two size descriptors among adult male athletes in combat sports (n = 64).

log a + k_1_ × log (X_1_) + k_2_ × log (X_2_) + log ε							Model Summary	
Y_i_: Performance Outputs	Constant		X_i_: Size Descriptors	k Exponent		Partial Correlation	R	R^2^ Adjusted
	Value	p		value	(95% CI)			
WAnT-PP	2.736	≤0.01	X_1_: FFM	0.800	0.307 to 1.293	0.384	0.742	0.550
WAnT-MP	3.474	≤0.01	X_1_: FFM	0.316	−0.024 to 0.656	0.232	0.804	0.647

95% CI (95% confidence interval); WAnT-PP (Wingate peak output); WAnT-MP (Wingate mean output); FFM (fat-free mass); LL-LST (Lower limbs lean soft tissue); a (constant); k (beta exponent); R (multiple regression coefficient); R^2^ (adjusted coefficient of determination).

**Table 4:** WANT test performance of male adult athletes in different combat sports in the current study and in other studies.

		n	CA (Years)	Stature (cm)	BM (kg)	FM		WAnT-PP		WAnT-MP	
						%	Technique	(watt)	(watt.kg^−1^)	(watt)	(watt.kg^−1^)
Present study	all	64	24 ± 5	174.3 ± 7.2	73.6 ± 12.1	12.7 ± 7.0	ADP	923 ± 166		696 ± 83	
	Boxing	11	26 ± 4	178.2 ± 7.1	78.3 ± 15.3	16.2 ± 8.4	ADP	890 ± 134		627 ± 100	
	Kickboxing	10	23 ± 3	173.9 ± 6.8	71.3 ± 10.1	9.2 ± 2.3	ADP	970 ± 135		608 ± 78	
	Taekwondo	9	23 ± 3	174.5 ± 6.5	75.7 ± 14.8	16.8 ± 9.9	ADP	907 ± 146		601 ± 86	
	Karate	9	22 ± 2	176.2 ± 6.5	71.2 ± 8.4	10.6 ± 7.0	ADP	1036 ± 112		650 ± 66	
	Judo	10	22 ± 2	173.5 ± 5.7	74.1 ± 10.7	7.5 ± 4.1	ADP	1014 ± 212		598 ± 60	
	Wrestling	5	25 ± 8	163.0 ± 6.4	62.2 ± 9.2	12.9 ± 3.3	ADP	743 ± 174		507 ± 99	
	Jiu-Jitsu	10	28 ± 6	174.9 ± 6.7	76.5 ± 11.5	15.5 ± 4.8	ADP	828 ± 105		604 ± 68	
Popadic Gacesa et al. [4]	Boxing	14	22.2	179.5	77			715	9.3	517	6.7
Zabukovec & Tiidus [23]	Kickboxing	4	27	176.7	72.6			1360	18.8	761	10.5
Taaffe & Pieter [22]	Taekwondo	14			72.5	7.5	Skinfolds	865	11.8	671	9.2
Doria et al. [20]	Karate (Kata)	3	30.7	176	78.5				9.7		5.7
	Karate (Kumite)	3	24	181	76.3				9.6		7.8
Sbriccoli et al. [21]	Judo	6	26	180	109			1236		558	
Popadic Gacesa et al. [4]	Wrestling	17	20.6	175.4	79.4			765	9.9	516	6.6
Yoon [24]	Wrestling	8							11.2		6.7
Lovell et. [25]	Jiu-Jitsu	1	25	182	90.2	8.5	Skinfolds	798	8.9	521	5.8

CA (chronological age); BM (body mass); FM (fat mass); ADP (air displacement plethysmography); WAnT-PP (Wingate peak power output); WAnT-MP (Wingate mean power output).

## References

[1] Green S.A. (1994). Definition and systems view of anaerobic capacity. Eur. J. Appl. Physiol. Occup. Physiol..

[2] Winter E.M., MacLaren D.P., Eston R.G., Reilly T. (2001). Assessment of maximal-intensity exercise. Kinanthropometry and Exercise Physiology Laboratory Manual: Tests, Procedures and Data.

[3] Khanna G.L., Manna I. (2006). Study of physiological profile of Indian boxers. J. Sports Sci. Med..

[4] Gacesa J.Z.P., Barak O.F., Grujic N.G. (2009). Maximal anaerobic power test in athletes of different sport disciplines. J. Strength Cond. Res..

[5] Bridge C.A., da Silva Santos J.F., Chaabene H., Pieter W., Franchini E. (2014). Physical and physiological profiles of taekwondo athletes. Sports Med..

[6] Ayalon A., Inbar O., Bar-Or O., Nelson R.C., Morehouse C.A. (1974). Relationships among measurements of explosive strength and anaerobic power. Biomechanics IV.

[7] Bar-Or O. (1987). The Wingate anaerobic test. An update on methodology, reliability and validity. Sports Med..

[8] Inbar O., Bar-Or O., Skinner J.S. (1996). The Wingate Anaerobic Test.

[9] Van Praagh E., Dore E. (2012). Short-term muscle power during growth and maturation. Sports Med..

[10] Tanner J.M. (1949). Fallacy of per-weight and per-surface area standards, and their relation to spurious correlation. J. Appl. Physiol..

[11] Nevill A.M., Ramsbottom R., Williams C. (1992). Scaling physiological measurements for individuals of different body size. Eur. J. Appl. Physiol. Occup. Physiol..

[12] Carvalho H.M., Coelho-e-Silva M.J., Figueiredo A.J., Gonçalves C.E., Phillippaerts R.M., Castagna C., Malina R.M. (2011). Predictors of maximal short-term power outputs in basketball players 14–16 years. Eur. J. Appl. Physiol..

[13] Nevill A.M., Holder R.L. (1994). Modelling maximum oxygen uptake: A case-study in non-linear regression model formulation and comparison. J. R. Stat. Soc. Ser. C Appl. Stat..

[14] Nevill A.M., Stewart A.D., Olds T., Holder R. (2004). Are adult physiques geometrically similar? The dangers of allometric scaling using body mass power laws. Am. J. Phys. Anthrop..

[15] Valente-dos-Santos J., Coelho-e-Silva M.J., Castanheira J., Machado-Rodrigues A.M., Cyrino E.S., Sherar L.B., Esliger D.W., Elferink-Gemser M.T., Malina R.M. (2015). The effects of sports participation on the development of left ventricular mass in adolescent boys. Am. J. Hum. Biol..

[16] Lohmann T.G., Roche A.F., Martorell R. (1998). Anthropometric Standardization Reference Manual.

[17] Coelho-e-Silva M.J., Rebelo-Gonçalves R., Martinho D., Ahmed A., Luz L.G.O., Duarte J.P., Severino V., Baptista R.C., Valente-dos-Santos J., Vaz V. (2018). Reproducibility of estimated optimal peak output using a force-velocity test on a cycle ergometer. PLoS ONE.

[18] Siri W.E. (1993). Body composition from fluid spaces and density: Analysis of methods. Nutrition.

[19] Hopkins W.G., Marshall S.W., Batterham A.M., Hanin A.J. (2009). Progressive statistics for studies in sports medicine and exercise science. Med. Sci. Sports Exerc..

[20] Sardinha L.B., Santos D.A., Silva A.M., Coelho-e-Silva M.J., Raimundo A.M., Moreira H., Santos R., Vale S., Baptista F., Mota J. (2012). Prevalence of overweight, obesity, and abdominal obesity in a representative sample of Portuguese adults. PLoS ONE.

[21] Doria C., Veicsteinas A., Limonta E., Maggioni M.A., Aschieri P., Eusebi F., Fanò G., Pietrangelo T. (2009). Energetics of karate (kata and kumite techniques) in top-level athletes. Eur. J. Appl. Physiol..

[22] Sbriccoli P., Bazzucchi I., Di Mario A., Marzattinocci G., Felici F. (2007). Assessment of maximal cardiorespiratory performance and muscle power in the Italian Olympic judoka. J. Strength Cond. Res..

[23] Taaffe D., Pieter W. (1990). Physical and physiological characteristics of elite taekwondo athletes. Commonwealth and International Conference Proceedings.

[24] Zabukovec R., Tiidus P.M. (1995). Physiological and anthropometric profile of elite kickboxers. J. Strength Cond. Res..

[25] Yoon J. (2002). Physiological profiles of elite senior wrestlers. Sports Med..

[26] Lovell D.I., Bousson M., McLellan C.A. (2013). The Use of Performance Tests for the Physiological Monitoring of Training in Combat Sports: A Case Study of a World Ranked Mixed Martial Arts Fighter. J. Athl. Enhanc..

[27] Jaafar H. (2017). Allometric scaling of power-force-velocity ergometry profiles in men. Ann. Hum. Biol..

[28] Lolli L., Batterham A.M., Weston K.L., Atkinson G. (2017). Size Exponents for scaling maximal oxygen uptake in over 6500 humans: A systematic review and meta-analysis. Sports Med..

[29] Hubner-Wozniak E., Kosmol A., Lutoslawska G., Bem E.Z. (2004). Anaerobic performance of arms and legs in male and female free style wrestlers. J. Sci. Med. Sport.

[30] Demirkan E., Koz M., Kutlu M., Favre M. (2015). Comparison of physical and physiological profiles in elite and amateur young wrestlers. J. Strength Cond. Res..

[31] Chaabene H., Negra Y., Bouguezzi R., Mkaouer B., Franchini E., Julio U., Hachana Y. (2017). Physical and physiological attributes of wrestlers: An update. J. Strength Cond. Res..

[32] Bussweiler J., Hartmann U. (2012). Energetics of basic karate kata. Eur. J. Appl. Physiol..

[33] Nevill A.M., Holder R.L., Baxter-Jones A., Round J.M., Jones D.A. (1998). Modeling developmental changes in strength and aerobic power in children. J. Appl. Physiol..

[34] McMahon T. (1973). Size and shape in biology. Science.

[35] Reljic D., Hassler E., Jost J., Friedmann-Bette B. (2013). Rapid weight loss and the body fluid balance and hemoglobin mass of elite amateur boxers. J. Athl. Train..

[36] Giovani N., Nicolaidis P. (2012). Differences in force-velocity characteristics of upper and lower limbs of non-competitive male boxers. Int. J. Exerc. Sci..

[37] Chaabène H., Tabben M., Mkaouer B., Franchini E., Negra Y., Amara S., Chaabène R.B., Hachana Y. (2015). Amateur boxing: Physical and physiological attributes. Sports Med..

[38] Vandewalle H., Peres G., Monod H. (1987). Standard anaerobic exercise tests. Sports Med..

[39] Jaafar H., Rouis M., Attiogbe E., Vandewalle H., Driss T.A. (2016). Comparative study between the wingate and force-velocity anaerobic cycling tests: Effect of physical fitness. Int. J. Sports Physiol. Perform..

